# "First, Do No Harm": Have the Health Impacts of Government Bills on Tax Legislation Been Assessed in Finland?

**DOI:** 10.15171/ijhpm.2018.39

**Published:** 2018-04-25

**Authors:** Natassa Aaltonen, Miisa Chydenius, Lauri Kokkinen

**Affiliations:** ^1^Faculty of Social Sciences, University of Tampere, Tampere, Finland.; ^2^Bloomberg Faculty of Nursing, University of Toronto, Toronto, ON, Canada.; ^3^Finnish Institute of Occupational Health, Tampere, Finland.

**Keywords:** Health in All Policies, Healthy Public Policy, Inter-sectoral Action, Taxation, Finland

## Abstract

As taxation is one of the key public policy domains influencing population health, and as there is a legal, strategic, and programmatic basis for health impact assessment (HIA) in Finland, we analyzed all 235 government bills on tax legislation over the years 2007–2014 to see whether the health impacts of the tax bills had been assessed. We found that health impacts had been assessed for 13 bills, bills dealing with tobacco, alcohol, confectionery, and energy legislation and that four of these impact assessments included impacts on health inequalities between social classes. Based on our theoretical classification, the health impacts of 40 other tax bills should have been evaluated.

## Background

Health impact assessment (HIA) is a tool for planning and decision making that helps to evaluate the impact of public policies on population health.^1^ In Finland, there is a legal basis for HIA, found in the Finnish Constitution (731/1999) and complemented by clarifications in the Local Government Act (2015/410), the Health Protection Act (1994/763), the Public Health Act (66/1972), and the Health Care Act (1326/2010). Furthermore, policy making in Finland is also guided by the European Union (EU), where the legal basis for HIA can also be found, especially in the Treaty on the Functioning of the EU (2012/C326/01) and in the Charter of Fundamental Rights of the EU (2012/C326/02).

In addition to the legal basis, the Finnish HIA mandate is complemented by guidelines, strategies, and programmes. Impact assessment guidelines for all policy sectors were introduced in 2007,^2^ and they follow the EU’s Better Regulation program.^3^ Many Finnish strategies and programs have aimed to develop the implementation of the HIA, such as the National Health 2015 – Public Health Program (effective 2001–2015), which introduced an extension of HIA for the preparation and re-evaluation of all public policies.^4^

Taxation is one of the key public policy domains influencing population health as taxation affects several social determinants of health, such as income and wealth distribution, employment, social protection, public services, and housing and living conditions.^5^ In Finland, it has been argued that the Ministry of Finance, and especially its tax department, has been resistant to assessing the impacts of the ministry’s decisions on health,^6^ but there is no empirical evidence for this claim. Therefore, we ask, have the health impacts of government bills on tax legislation been assessed in Finland?

## Methods

We chose the government’s taxation bills as the study data, because the government represents the supreme level of public authority; the government’s legislative bills reflect the commitment to the government’s program and to other health policy guidelines during the government term. Furthermore, the content of government bills is important as most of the propositions that pass the preparation stage are accepted in the Finnish Parliament as they are or with minor technical or grammatical modifications.^7^ Our data consist of government bills on tax legislation from 2007 to 2014. We chose an eight-year period to overcome yearly fluctuation, and the data were available until the end of 2014. The data cover altogether 235 tax bills, which are all freely available from the Parliament of Finland’s website https://www.eduskunta.fi/FI/lakiensaataminen/valtiopaivaasiat/Sivut/hallituksen-esitykset.aspx (in Finnish only).

We analyzed the data with theory-driven content analysis. The analysis was based on previous understanding regarding the social determinants that are root causes of population health and health inequalities. We divided these bills into two categories. Category I consists of bills that based on previous literature have a considerable impact on population health. This category covers bills that deal with income distribution, housing, employment, lifestyle, addictive substances (eg, alcohol and tobacco consumption), energy, health insurance, social security, inheritance tax, and gift tax.^5,8^ All other bills we placed in category II. After classifying the data, we calculated the number of bills in each category and the number of HIAs made during 2007–2010 and 2011–2014.

## Results

As shown in Table, of the 235 government bills on tax legislation the health impacts had been assessed for 13 (6% of all tax bills). Four of these impact assessments also included an assessment of health inequalities between social classes.

**Table:** HIAs of Government Bills on Tax Legislation in Finland From 2007 to 2014

**Category**		**I**	**II**	**Total**
Government period 2007–2010	Government bills	27	114	141
	HIA implemented	7	0	7
Government period 2011–2014	Government bills	26	68	94
	HIA implemented	6	0	6
Total number of bills		53	182	235
Total number of HIAs		13	0	13

Abbreviation: HIA, health impact assessment.

During the 2007–2010 term, 26% of the tax bills that we categorized as potential health determinants had an HIA done. The same was true of 23% of the bills in the 2011–2014 term. This means that we observed no positive trend in the frequency of HIA despite the strengthened legal basis and the launch of HIA guidelines, strategies, and programs.

Based on our theoretical classification, the health impacts of 40 other tax bills should have been evaluated. Health impacts were assessed in some of the bills related to tobacco, alcohol, confectionery, and energy legislation and mainly concerned consumption. However, health impacts were not assessed in any of the bills related to income distribution, housing, employment, health insurance, social security, inheritance tax, and gift tax, even if tax proposals in each of these categories were present in the data.

Among the 235 tax bills, environmental impacts were assessed more often than health impacts, and economic impacts were assessed in almost all bills.

## Discussion

In this study, we found that health impacts had been assessed for 13 bills dealing with tobacco, alcohol, confectionery, and energy legislation and that based on our theoretical classification, the health impacts of 40 other tax bills should have been evaluated.

It is not surprising that most of the HIAs we found dealt with addictive substances. There is a relatively strong scientific evidence base for these downstream determinants of health, and there is strong previous experience in Finland in evaluating the health impacts of alcohol and tobacco policies.^9^ On the other hand, the evidence base for structural determinants of health (eg, income and wealth distribution) is more debatable: There is less experience in Finland of detecting and evaluating their contribution to population health, and evidence of these upstream health determinants is often in conflict with someone’s vested interests or political ideology.^10^

The legislation and guidance that mandate HIA in Finland do not name any specific health determinants. Specifying the structural determinants of health within the mandate might help to make the impacts of upstream determinants of health visible. This does not mean that HIAs would automatically and immediately lead to changes being made in the policies, as recent evidence suggests that even when the health impacts have been assessed as negative and large, other impacts, such as positive economic ones, may have carried more weight for decision making in the Finnish Parliament.^11,12^ However, in the longer term a mandate specifying HIA of structural determinants of health should help to increase the transparency of policy processes and make the policy-makers more responsible for their decisions. ^.^

As Finland is one of the rare countries where HIAs are mandated for national policy proposals, there is not much previous research in this area despite the critical role taxation and other key public policy domains play in population health through a wide range of social determinants of health. Therefore, as more countries currently adopt a Health in All Policies strategy and institutionalize related techniques such as HIAs,^12^ there is a great need for researchers to understand the importance of HIAs for different public policy domains, to gain experience in the focus of HIA on downstream and upstream determinants of health, as well as to ultimately use science to achieve greater government transparency in taking health into account in all policies.

## Ethical issues

Not applicable.

## Competing interests

Authors declare that they have no competing interests.

## Authors’ contributions

NA was involved in designing the study, collecting the data and drafting the manuscript for content, including analysis and interpretation of data. MC was involved in designing the study, collecting and analyzing the data and drafting the manuscript for content. LK was involved in designing the study and critically revising the manuscript for important intellectual content. All authors have approved the final version of the manuscript and agree to be accountable for all aspects of the work.

## Authors’ affiliations

^1^Faculty of Social Sciences, University of Tampere, Tampere, Finland. ^2^Bloomberg Faculty of Nursing, University of Toronto, Toronto, ON, Canada. ^3^Finnish Institute of Occupational Health, Tampere, Finland.

## References

[1] Kemm J, Parry J. What is HIA? Introduction and overview. In: Kemm J, Parry J, Palmer S, eds. Health Impact Assessment. New York: Oxford University Press; 2004:1-13.

[2] Ministry of Justice. Better Regulation. Helsinki: Edita; 2007.

[3] European Commission. Better Regulation - Simply Explained. Luxembourg: Office for Official Publications of the European Communities; 2006.

[4] Ministry of Social Affairs and Health. Government Resolution on the Health 2015 Public Health Programme. Helsinki: Ministry of Social Affairs and Health; 2001.

[5] Commission on Social Determinants of Health. Closing the gap in a generation: Health equity through action on the social determinants of health. Geneva: World Health Organization; 2008. 10.1016/S0140-6736(08)61690-6 18994664

[6] Stahl T, Rimpela A. Terveyden edistäminen tutkimuksen ja päätöksenteon haasteena. Helsinki: Yliopistopaino; 2010.

[7] Ervasti K, Tala J, Castren E. Lainvalmistelun laatu ja eduskunnan valiokuntatyo. Helsinki: Oikeuspoliittinen tutkimuslaitos; 2000.

[8] Sihto M, Palosuo H, Topo P, Vuorenkoski L, Leppo K. Terveyspolitiikan perusta ja käytännöt. Helsinki: Terveyden-ja hyvinvoinnin laitos; 2013.

[9] Stahl T, Wismar M, Ollila E, Lahtinen E, Leppo K. Health in All Policies: Prospects and potentials. Helsinki: Ministry of Social Affairs and Health; 2006.

[10] Kokkinen L, Shankardass K, O’Campo P, Muntaner C (2017). Taking health into account in all policies: raising and keeping health equity high on the political agenda. J Epidemiol Community Health.

[11] Kokkinen L, Muntaner C, O’Campo P, Freiler A, Oneka G, Shankardass K (2017). Implementation of Health 2015 public health program in Finland: a welfare state in transition. Health Promot Int.

[12] Shankardass K, Muntaner C, Kokkinen L (2018). The implementation of Health in All Policies initiatives: a systems framework for government action. Health Res Policy Syst.

